# Decoupling of nitrogen allocation and energy partitioning in rice after flowering

**DOI:** 10.1002/ece3.11297

**Published:** 2024-04-15

**Authors:** Duwei Zhong, Yonggang Chi, Jianxi Ding, Ning Zhao, Linhui Zeng, Pai Liu, Zhi Huang, Lei Zhou

**Affiliations:** ^1^ College of Geography and Environmental Sciences Zhejiang Normal University Jinhua China; ^2^ Key Laboratory of Ecosystem Network Observation and Modeling, Institute of Geographic Sciences and Natural Resources Research Chinese Academy of Sciences Beijing China

**Keywords:** flowering, fluorescence yield (ΦF), leaf energy partitioning, leaf nitrogen allocation, photochemical yield (ΦP)

## Abstract

Estimation of energy partitioning at leaf scale, such as fluorescence yield (ΦF) and photochemical yield (ΦP), is crucial to tracking vegetation gross primary productivity (GPP) at global scale. Nitrogen is an important participant in the process of light capture, electron transfer, and carboxylation in vegetation photosynthesis. However, the quantitative relationship between leaf nitrogen allocation and leaf energy partitioning remains unexplored. Here, a field experiment was established to explore growth stage variations in energy partitioning and nitrogen allocation at leaf scale using active fluorescence detection and photosynthetic gas exchange method in rice in the subtropical region of China. We observed a strongly positive correlation between the investment proportion of leaf nitrogen in photosynthetic system and ΦF during the vegetative growth stage. There were significant differences in leaf energy partitioning, leaf nitrogen allocation, and the relationship between ΦF and ΦP before and after flowering. Furthermore, flowering weakened the correlation between the investment proportion of leaf nitrogen in photosynthetic system and ΦF. These findings highlight the crucial role of phenological factors in exploring seasonal photosynthetic dynamics and carbon fixation of ecosystems.

## Graphical abstract

Significant differences in leaf scale nitrogen allocation and leaf scale energy partitioning before and after flowering. The linear relationship between fluorescence yield and photochemical yield decouples during the reproductive growth stage. Fluorescence yield during vegetative growth can track the investment of leaf nitrogen in the photosynthetic system.

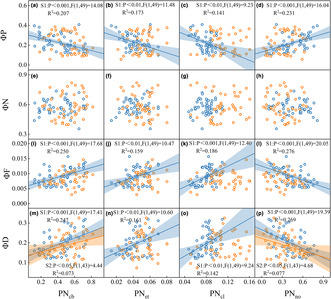

## 1 INTRODUCTION

Sun‐induced chlorophyll fluorescence (SIF) is commonly used to track vegetation carbon assimilation at multiple scales (Frankenberg et al., 2011; Guanter et al., 2014; Maguire et al., 2020; Yang et al., 2015). At global scale, SIF has been found strongly linear correlated with gross primary productivity (GPP) based on satellite measurements of OCO‐2, GOSAT and GOME‐2 (Guanter et al., 2012; Sun, Frankenberg, et al., 2018; Zhang et al., 2016). Additionally, using satellite measurements from TROPOMI, it has also been observed that the relationship between SIF and GPP exhibits varying degrees of non‐linearity due to the saturation of GPP at moderate to high SIF levels (Balde et al., 2023). At canopy scale, SIF has become a good proxy for tracking seasonal dynamics of vegetation photosynthetic capacity (Campbell et al., 2019; Magney et al., 2019; Walther et al., 2016; Yang et al., 2017). At leaf scale, fluorescence yield (ΦF) and photochemical yield (ΦP) exhibit a distinct nonlinear relationship with light intensity, characterized by a “boomerang” pattern, when measured at high temporal resolution (Zhang et al., 2016). However, the challenge in utilizing SIF to track seasonal dynamics of carbon assimilation still lies in the physiological limitations of decoding PSII photochemical yield from SIF (Magney et al., 2019; Maguire et al., 2020). The relationship between ΦF and ΦP reflects a series of complex electron transfer processes involved in both light reactions and dark reactions in PSII (Mlinaric et al., 2021). Therefore, it is necessary to further investigate the process of leaf energy partitioning.

Energy partitioning in photosystem II, which is largely driven by absorbed photosynthetically active radiation (APAR) at diurnal timescales (Baker, 2008; van der Tol et al., 2016; Yang et al., 2015), is regulated by seasonal variations of leaf nitrogen allocation (Martini et al., 2019). Photon captured by chlorophyll drive three competitive processes: photochemical reactions, heat, and chlorophyll fluorescence (Alonso et al., 2017; Hmimina et al., 2014; Stirbet et al., 2020; van der Tol et al., 2014; Xu et al., 2021). Under low irradiance, the majority of absorbed energy is utilized for photochemical reactions, leading to lower ΦF and suppressed non‐photochemical quenching (NPQ) (Baker, 2008). As irradiance increases, electron transport chain turns into saturated gradually, resulting in a decrease in ΦP and an increase in ΦF (Porcar‐Castell et al., 2014). Under saturated irradiance, the excessive APAR at the NPQ‐limit dissipates mainly in the form of heat, leading to a positive relationship between ΦP and ΦF (Maguire et al., 2020). Nevertheless, variations in leaf nitrogen allocation across different growth stages can potentially modulate energy partitioning processes (Ač et al., 2015; Kalaji et al., 2016; Mishra et al., 2019; Mu et al., 2017). Leaf nitrogen within the photosynthetic apparatus is divided into three distinct categories: carboxylation system (N_cb_), bioenergetic protein (N_et_), and light‐harvesting protein (N_cl_), with the rest going to non‐photosynthetic components (N_no_) (Bahar et al., 2017; Ghimire et al., 2017; Hikosaka, 2004; Hikosaka & Shigeno, 2009; Tang et al., 2018). Under nitrogen stress, crops tend to allocate more nitrogen to bioenergetic protein (PN_et_) and less nitrogen to light‐harvesting proteins (PN_cl_) (Mu et al., 2018; Mu & Chen, 2021). Although previous studies have confirmed that leaf nitrogen concentration modulates light absorbance and SIF emission (Martini et al., 2019; Mu et al., 2017, 2018; Mu & Chen, 2021), the quantitative relationship between nitrogen allocation and energy partitioning has yet to be determined.

Flowering regulates leaf nitrogen allocation and leaf energy partitioning (Rios et al., 2017; Simko et al., 2020; Sun et al., 2020). Approximately 75% of the leaf nitrogen accumulated during the vegetative growth stage was subsequently transferred to crop seeds during the reproductive growth stage of winter wheat (Pask et al., 2012). Significant differences in rice's maximum rate of carboxylation (*V*_cmax_) were found across various growth stages, under both ambient CO_2_ and elevated CO_2_ concentration (Yang et al., 2021). During the transition from the vegetative growth to the flowering, leaves exhibit significant differences in photosynthetic rates by increasing resource uptake and utilization (i.e., enhancing photosynthetic efficiency) (Tang et al., 2021). In addition, Yang et al. (2018) reported a significant positive relationship between ΦF and ΦP existed during the vegetative growth stage, while decoupled diurnal variations of ΦP and ΦF were observed during the mature stage. Sun et al. (2020) found that the ratio of canopy far‐red band fluorescence to red band fluorescence during the early growth stage of oilseed rape was significantly higher compared to the mature stage. Thus, it is necessary to investigate the impacts of flowering on the relationship between leaf nitrogen allocation and leaf energy partitioning.

Rice is one of the world's most important crops and serves as a vital food source for over half of the global population (Xue et al., 2021; Zeng et al., 2017). However, global food security faces significant threats from global warming, extreme climate events, industrialization, pests, and diseases (Challinor et al., 2014; Schiferl et al., 2018). The relationship between leaf nitrogen allocation and leaf energy partitioning is critical to accurately monitor GPP of crops using SIF. Here, a field experiment was conducted to investigate leaf nitrogen allocation and leaf energy partitioning using active fluorescence detection and photosynthetic gas exchange measurements in rice during vegetative and reproductive growth stages in the subtropical region of China. Our objectives are to explore (1) whether the energy partitioning at leaf scale is regulated by leaf nitrogen allocation and (2) whether the relationship between leaf nitrogen allocation and leaf energy partitioning is affected by flowering.

## 2 MATERIALS AND METHODS

### 2.1 Study site description

The study was conducted at Shangshan Rice Research Station in Zhejiang province, located in South China. The region was characterized as typical subtropical monsoon climate, with a mean annual temperature of 16.4°C and a mean annual precipitation of 1450 mm (Wang et al., 2022). Meanwhile, mean annual relative humidity is 79% and mean total sunshine is 1996 h (Xu et al., 2014; Zheng et al., 2019). The seasonal distribution of precipitation is uneven, with abundant rainfall in spring and during the monsoon season. Approximately 54% of the annual rainfall occurs from March to June (Huang et al., 2016). The soil composition in this region mainly consists of red soil, yellow soil, lithologic soil, tidal soil, and paddy soil (Zhi et al., 2015). The rice paddy was planted at a density of 16 plants m^−2^ and maintained according to the standard agronomic practice of the region.

### 2.2 Experimental design

The field experiments were conducted in three blocks in 2021. Each block had three treatments, namely control group (N0, no artificial nitrogen application), the middle nitrogen group (N1, 11.73 g N m^−2^) and the high nitrogen group (N2, 23.46 g N m^−2^). In our study, N1 represented the typical agronomic fertilization pattern in the study region, while N0 and N2 referred to the low N and high N treatments, respectively. A total of nine quadrats were established with a cement board to prevent nitrogen loss. The application of urea fertilizer followed a specific ratio of 4:3:3 at the tree key growth stages: tillering (DOY 175), jointing (DOY 200), and heading (DOY 254). Additionally, phosphate (1.536 g P m^−2^) and potash (12.6 g K m^−2^) fertilizers were managed according to farmland fertilization standards. Field measurements were carried out 10 times, spaced approximately 7–10 days apart, during the period from DOY198 to DOY287 within 1 year. In the ecosystem, graminoid plants typically delay flowering and reduce reproductive allocation in response to nitrogen addition (Niu et al., 2008; Zhang, Niu, et al., 2014). In our study, the flowering periods of rice were concentrated from DOY 240 to 251 under different nitrogen treatments.

### 2.3 Leaf gas exchange measurements

Photosynthetic assimilation versus intercellular CO_2_ response (*A*_n_/*C*_i_) curves were measured using an Li‐6800 portable steady‐state photosynthesis system (LI‐COR Inc., USA) on a clear and sunny day between 8:00 a.m. and 12:00 p.m. We utilized a Li‐190R quantum sensor carried by Li‐6800 to measure the incident photosynthetic radiation of leaves (PAR_leaf_) simultaneously. The fully expanded leaves were measured under saturating photosynthetic photon flux density (PPFD) of 1500 μmol m^−2^ s^−1^ and relative air humidity of 55%. The carbon dioxide concentration of the reference chamber was varied sequentially, with values set at 400, 300, 200, 100, 50, 400, 600, 800, 1000, and 1200 μmol CO_2_ mol^−1^. Before measurements, the leaves were acclimated for 5 min at saturated light, ambient temperature, and a CO_2_ concentration of 400 μmol CO_2_ mol^−1^. Net photosynthesis (*A*_n_), air temperature (*T*_air_), leaf temperature (*T*_leaf_), and vapor pressure deficit (VPD) were logged synchronously. A total of 90 data points (i.e., 3 nitrogen levels × 3 quadrats per nitrogen levels × 10 times = 90 data) were collected throughout vegetative and reproductive growth stages. Concurrently, an ASD FieldSpec 4 spectrometer (ASD Inc., USA) was used to synchronously record the reflectance spectra of leaves at 350–2500 nm, with a spectral resolution of 1 nm (Note S1).

### 2.4 Chlorophyll fluorescence measurements

Chlorophyll fluorescence parameters at leaf scale were measured using a mini‐PAM‐II fluorometer (Heinz Walz GmbH Inc., Germany). In the pulse amplitude modulation (PAM) technique, chlorophyll fluorescence was measured using two light sources: one to drive photosynthesis and the other to excite fluorescence. When background actinic or saturating illumination was present, the measuring flashes were applied at a high frequency to capture the rapid fluorescence changes induced by the actinic/saturating light. To eliminate any potential influence of background light, which could affect the accuracy of the modulated fluorescence readings, measurements were taken immediately after the measuring flash. This precaution was particularly important when conducting measurements in the field. We measured the dark adaptation chlorophyll fluorescence parameters of rice leaves after sunset on the first day of the experimental cycle (PPFD = 0). Meanwhile, the light adaptation fluorescence parameters of rice leaves were measured during two time periods from 6:00 to 9:00 a.m. and 9:00 to 12:00 p.m., when was the rising stage of irradiance and photosynthetic capacity to avoid photosynthetic inhibition and plant photodamage in the afternoon. During the measurement, we employed a weak measuring light intensity of 0.5 μmol m^−2^ s^−1^ to assess the minimum fluorescence levels. Subsequently, a saturation pulse of 8000 μmol m^−2^ s^−1^, lasting approximately 1 s, we applied to measure the maximum fluorescence. The measured parameters encompassed the following: the minimal fluorescence yield at the dark‐adapted state (*F*_0_), the maximal fluorescence yield at the dark‐adapted state (*F*_m_), the maximal quantum yield of PSII photochemistry (*F*_v_/*F*_m_), the maximal fluorescence yield of the light adapted state ($F_{m}^{\prime }$), the steady state fluorescence yield (*F*_s_), the actual photochemical efficiency of PSII (ΦP), and the actual electron transport rate (ETR, μmol m^−2^ s^−1^).

### 2.5 Leaf nitrogen and chlorophyll content measurements

Leaves close to those used in the measurements of *A*_n_/*C*_i_ curve were cut out to measure chlorophyll per leaf mass (Chl_mass_). The leaf samples of mass 0.1 g were cut into filaments and placed in the test tube containing a mixture of acetone and absolute ethanol. The volume of acetone and absolute ethanol was 1:1. The test tube was placed in a cool and dark environment to minimize the influence of light and temperature on the chlorophyll exaction (Zhuang et al., 2021). The absorbance of chlorophyll at wavelength of 645 and 663 nm was measured with a spectrophotometer when the leaf filaments were completely white. The chlorophyll content was calculated as follows (Wellburn, 1994):

(1) $${\mathrm{Chl}}_{a}=12.72\times A663-2.59\times A645,$$

(2) $${\mathrm{Chl}}_{b}=22.88\times A645-4.67\times A663,$$

(3) $${\mathrm{Chl}}_{\text{mass}}={\mathrm{Chl}}_{a}+{\mathrm{Chl}}_{b},$$

where Chl refers to chlorophyll content, and subscripts a, b, and mass represent chlorophyll a, chlorophyll b, and total chlorophyll content (mg L^−1^), respectively. The *A*645 and *A*663 denote the absorbances at wavelengths of 645 and 663 nm, respectively.

Leaf mass per area (LMA, mg cm^−2^) was calculated as the ratio of leaf dry mass and leaf area. Fresh rice leaves were firstly dried in an oven at 105°C for 30 min and then dried at 80°C until constant weight was achieved. Leaves powder of ~5 mg was used for the determination of nitrogen content per leaf mass (N_mass_) using a Vario ELcube elemental analyzer (Elementar Inc., Germany). Subsequently, leaf nitrogen content per unit the leaf area (N_area_, mg cm^−2^) was calculated as N_area_ = N_mass_ × LMA.

### 2.6 Statistical analysis

The forms of nitrogen in plants are diverse (Note S2). Over the past two decades, leaf nitrogen has been mainly classified into two functional parts: nitrogen for the photosynthetic system and nitrogen for non‐photosynthetic components (Niinemets, 2007; Niinemets & Tenhunen, 1997; Novriyanti et al., 2012; Westbeek et al., 1999). Meanwhile, the allocation proportion of leaf nitrogen to photosynthetic system is generally divided into three components, including carboxylation system (PN_cb_, g g^−1^), bioenergetic protein (PN_et_, g g^−1^), and light‐harvesting protein components (PN_cl_, g g^−1^) (Zhuang et al., 2021). These components are calculated from maximum rate of carboxylation (*V*_cmax_), maximum rate of electron transfer (*J*_max_), and chlorophyll content (C_c_) following the below equations (Niinemets & Tenhunen, 1997):

(4) $${\mathrm{PN}}_{\mathrm{cb}}=\frac{V_{\text{cmax}}}{6.25\times V_{\mathrm{cr}}\times \mathrm{LMA}\times N_{\text{mass}}},$$

(5) $${\mathrm{PN}}_{\mathrm{et}}=\frac{J_{\max}}{8.06\times J_{\mathrm{mc}}\times \mathrm{LMA}\times N_{\text{mass}}},$$

(6) $${\mathrm{PN}}_{\mathrm{cl}}=\frac{C_{\mathrm{ab}}}{C_{b}\times N_{\text{mass}}},$$

(7) $${\mathrm{PN}}_{\mathrm{no}}=1-\left({\mathrm{PN}}_{\mathrm{cb}}+{\mathrm{PN}}_{\mathrm{et}}+{\mathrm{PN}}_{\mathrm{cl}}\right),$$

where PN_no_ (g g^−1^) is the investment proportion of leaf nitrogen in non‐photosynthetic system. *V*_cmax_ and *J*_max_ were calculated by fitting *A*_n_/*C*_i_ curves using the spreadsheet developed by Sharkey et al. (2007). The value of 6.25 is the conversion coefficient of rubisco enzyme to nitrogen and 8.06 is the number of cytochromes per gram of nitrogen in the bioenergy conversion carrier. *V*_cr_ represents the CO_2_ carboxylation activity of the unit rubisco enzyme with a value of 20.78 μmol CO_2_ (g rubisco)^−1^ s^−1^ at 25°C; *J*_mc_ represents the number of electrons transmitted per second by cytochrome with a value of 155.65 μmol electrons (μmol Cyt f)^−1^ s^−1^ at 25°C. C_ab_ is the chlorophyll content (mmol g^−1^); C_b_ is the content of the chlorophyll–protein complex with a value of 2.15 mmol g^−1^. When simulating the investment proportion of leaf nitrogen to non‐photosynthetic system (PN_no_), any negative values were adjusted to zero.

Heat dissipation is partitioned as the constitutive dark‐adapted thermal dissipation (D) and the energy‐dependent heat dissipation (N) under the light conditions. The yields of these dissipation processes were calculated using the following equations (Lee et al., 2015; van der Tol et al., 2014; Zhang et al., 2016):

(8) ΦP+ΦN+ΦF+ΦD=1,

(9) $$\mathrm{\Phi P}=\frac{K_{P}}{K_{P}+K_{N}+K_{F}+K_{D}},$$

(10) $$\mathrm{\Phi N}=\frac{K_{N}}{K_{P}+K_{N}+K_{F}+K_{D}},$$

(11) $$\mathrm{\Phi F}=\frac{K_{F}}{K_{P}+K_{N}+K_{F}+K_{D}},$$

(12) $$\mathrm{\Phi D}=\frac{K_{D}}{K_{P}+K_{N}+K_{F}+K_{D}},$$

(13) $$K_{D}=\mathrm{MAX}\left(0.03\times T+0.0773,0.87\right),$$

(14) $$K_{N}=\frac{F_{m}-F_{m}^{\prime }}{F_{m}^{\prime }}\times \left(K_{F}+K_{D}\right),$$

(15) $$K_{P}=\frac{F_{m}^{\prime }-F_{s}}{F_{s}}\times \left(K_{F}+K_{D}+K_{N}\right),$$

where Φ is the proportion of photosynthetic energy partitioning with subscripts P and F for photochemical reactions, fluorescence, respectively, and N and D for heat dissipation (the sum of ΦN and ΦD is the NPQ yield); *K*_F_ is the rate constant for fluorescence, taken as 0.05 (Lee et al., 2015; van der Tol et al., 2014); *K*_D_ is the rate coefficient of dark‐adapted thermal dissipation, which is a function of temperature (*T*) (Lee et al., 2015; van der Tol et al., 2014); *K*_N_ and *K*_P_ are the rate coefficients of energy‐dependent heat dissipation and photochemistry, respectively; *F*_m_ is the maximal fluorescence after dark adaptation; $F_{m}^{\prime }$ is the maximal fluorescence under the light condition; and *F*_s_ is the steady‐state fluorescence. ΦP, *F*_m_, $F_{m}^{\prime }$ and *F*_s_ can be measured directly by the mini‐PAM‐II fluorometer.

One‐way ANOVA was used to test the impact of nitrogen treatment on physiological parameters. If the effects of nitrogen treatment were significant, multiple comparisons were performed by Fisher's least significant difference (LSD) test at a significance level of *p* < .05. Two‐way ANOVA was used to test the effects of nitrogen, stage, and their interaction on leaf nitrogen allocation and leaf energy partitioning. A correlation matrix was used to conduct a two tailed test on nitrogen allocation components and energy partitioning percentage. The significance level was set at two levels: *p* < .05 and *p* < .01. Linear regressions were utilized to examine how leaf nitrogen allocation impacts on leaf energy partitioning at different growth stages, determined by the coefficient of determination (*R* ^2^) and *p*‐value. The distinction between non‐photochemical quenching at high irradiance (NPQ‐limited) and photochemical quenching at low irradiance (PQ‐limited) was determined by fitting the peaks of lines with second‐order polynomials (Figure S2). Statistical analysis was conducted using IBM SPSS 26.0 (SPSS, Chicago, IL, USA). Data visualization was performed using origin 2021 (Origin‐Lab, USA) and R Version 4.1.2.

## 3 RESULTS

### 3.1 Growth variation of leaf energy partitioning and physiological characteristics

**FIGURE 1:**
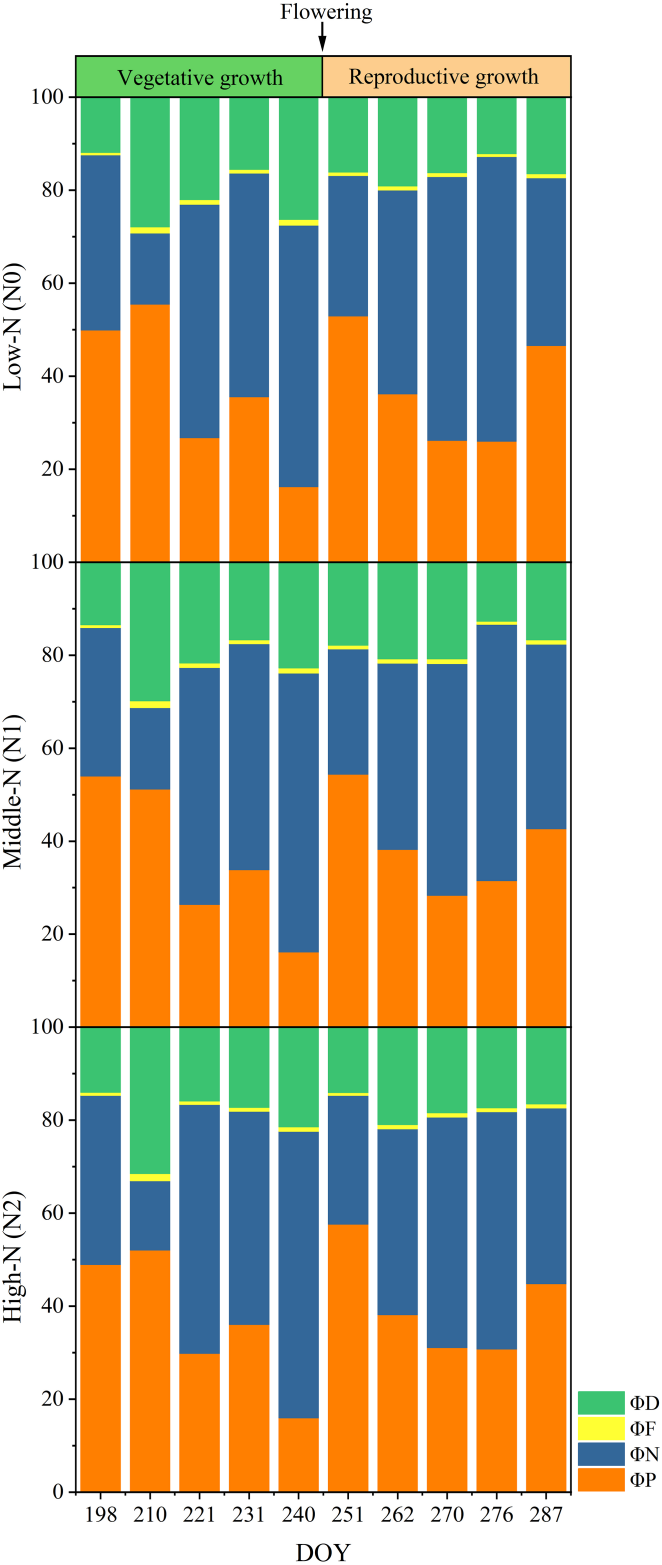
Growth variations in the quantum yields (Φ) for the different pathways: photosynthesis (ΦP, orange), nonphotochemical quenching (ΦN, blue), fluorescence (ΦF, yellow), and nonradiative decay (ΦD, green).

During the entire growth cycle, ΦN had the highest proportion with an average value of 0.42, followed by ΦP at 0.38, ΦD at 0.19, and ΦF at 0.01 (Figure 1, Table 1). Specifically, ΦF and ΦD were higher before flowering than that after flowering, while ΦP and ΦN exhibited the opposite trend (Table 1). Additionally, a negative correlation was observed between ΦF and ΦN both before flowering (*p* = .002, *R* = .399) and after flowering (*p* = .000, *R* = .303) (Figure S2).

**TABLE 1:** Means and standard errors of energy partitioning components under different growth stages and nitrogen treatments.

Stage	Nitrogen	ΦP	ΦN	ΦF	ΦD
S1	N0	0.37 ± 0.20 (a)	0.41 ± 0.19 (a)	0.010 ± 0.003 (a)	0.21 ± 0.07 (a)
	N1	0.36 ± 0.18 (a)	0.42 ± 0.19 (a)	0.010 ± 0.004 (a)	0.21 ± 0.07 (a)
	N2	0.37 ± 0.17 (a)	0.42 ± 0.20 (a)	0.010 ± 0.004 (a)	0.20 ± 0.07 (a)
S2	N0	0.37 ± 0.22 (a)	0.46 ± 0.21 (a)	0.008 ± 0.002 (a)	0.16 ± 0.05 (a)
	N1	0.39 ± 0.21 (a)	0.42 ± 0.20 (a)	0.009 ± 0.003 (a)	0.18 ± 0.06 (a)
	N2	0.40 ± 0.21 (a)	0.41 ± 0.19 (a)	0.008 ± 0.002 (a)	0.17 ± 0.05 (a)

*Note*: S1, vegetative growth stage; S2, reproductive growth stage; ΦD, nonradiative decay yield; ΦF, fluorescence yield; ΦN, non‐photochemical quenching yield; ΦP, photochemical yield. Values are means ± SE (*n* = 15). In the same column within a specific growth stage, different lowercase letters indicate significant differences between nitrogen treatments as determined by ANOVA (*p* < .05).

There was no significant difference in LMA before and after flowering (ANOVA *F*(1, 81) = 2.560, *p* = .122; Tables S1 and S2). The nitrogen content of leaves, represented by N_mass_ and N_area_, exhibited a consistent decreasing trend throughout the entire growth season (Figure S3c,e). Similarly, the values of *V*_cmax_, *J*_max_, and C_ab_ also showed a decreasing trend over the growing season (Figure S3b–f). Before flowering, *V*_cmax_, *J*_max_, and C_ab_ values were significantly higher compared to those after flowering (ANOVA *F*(1, 81) = 20.139, 73.760, 15.209; *p* < .01; Table S2).

### 3.2 Response of energy partitioning to environment change

**FIGURE 2:**
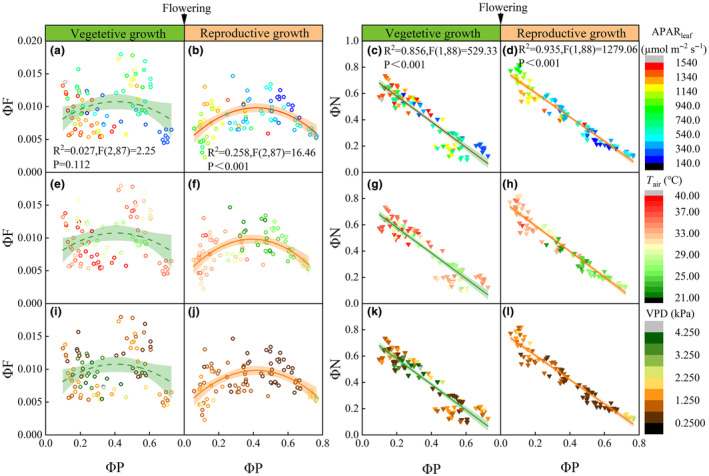
Relationships of ΦP (photochemical yield) with ΦF (fluorescence yield) and ΦN (non‐photochemical quenching) during vegetative growth (a, c, e, g, i, k) and reproductive growth stages (b, d, f, h, j, l). APAR_leaf_, photosynthetically active radiation absorbed by leaf; *T*_air_, air temperature; VPD, vapor pressure deficit. The left side is a second‐order polynomial fitting curve, and the right side is a linear fitting curve, the shaded area represents the 95% confidence interval of the fitting line. The color gradients on the right side of the graph represent the changes in APAR_leaf_, *T*_air_, and VPD, respectively.

ΦF (0.004–0.018) during the vegetative growth stage was significantly higher than that in the reproductive growth stage (0.003–0.015) (Figure 2). Notably, the average values of APAR_leaf_, *T*_air_ and VPD during the vegetative growth stage were 920 μmol m^−2^ s^−1^, 33°C and 1.7 kPa, respectively. These values exceeded those recorded during the reproductive growth stage, with an average of 702 μmol m^−2^ s^−1^ for APAR_leaf_, 29°C for *T*_air_, and 1.0 kPa for VPD. With the continuous increase of APAR_leaf_, there was a transition in PSII activity from photochemical quenching limited (PQ‐limited) to non‐photochemical quenching limited (NPQ‐limited). This transition point occurred at APAR_leaf_ value of 940 μmol m^−2^ s^−1^ during the vegetative growth stage and 540 μmol m^−2^ s^−1^ during the reproductive growth stage. Simultaneously, during the vegetative growth stage, when rice experienced PQ‐limited (ΦP > 0.41) (Figure S2), the sustained high values of *T*_air_ and VPD led to upward fluctuations and increase in ΦF (Figure 2).

### 3.3 Relationship of energy partitioning with N_mass_ and leaf nitrogen allocation

**FIGURE 3:**
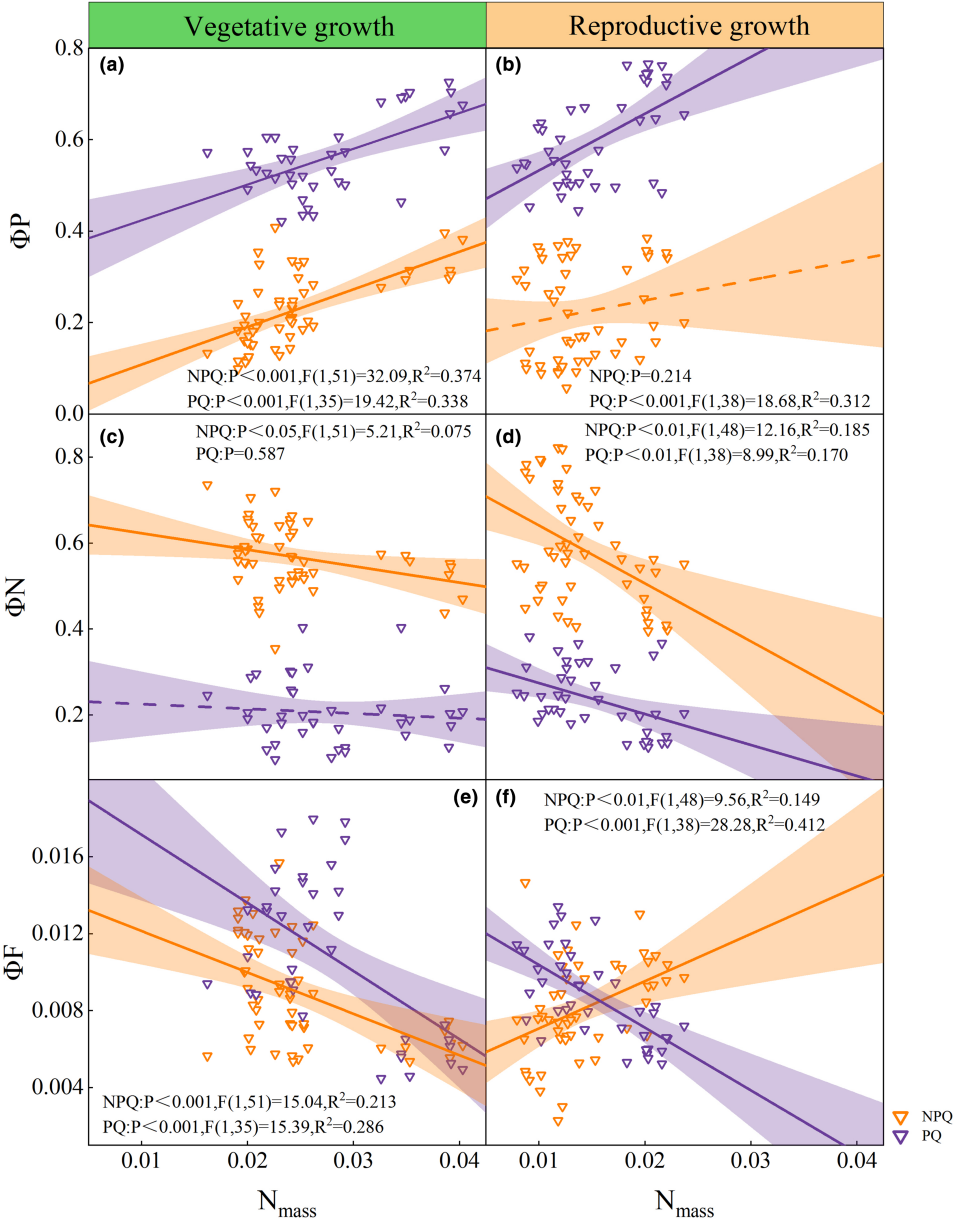
Relationship between N_mass_ (nitrogen per leaf mass) with energy partitioning: ΦP (photochemical yield), ΦN (non‐photochemical quenching yield), ΦF (fluorescence yield) during vegetative growth (a, c, e) and reproductive growth stages (b, d, f). Orange indicates that ΦF is limited by non‐photochemical quenching (NPQ‐limited), while purple indicates that ΦF is limited by photochemical quenching (PQ‐limited). The fitting line and 95% confidence interval are represented in the same color.

During the vegetative growth stage, an increase in nitrogen content per leaf mass (N_mass_) resulted in an enhancement in photochemical yield (ΦP), accompanied by a decrease in non‐photochemical quenching (ΦN) and fluorescence yield (ΦF) (Figure 3).

During the whole growth cycle, PN_cb_ showed the highest proportion at an average value of 0.49, followed by PN_no_ at 0.36, PN_cl_ at 0.10, and PN_et_ at 0.10 (Figure S4, Table S3). Significant differences in nitrogen allocation patterns were observed before and after flowering (ANOVA *F*(1, 79) = 7.341, 5.486, 62.081, 12.441; all *p* < .01; Figure S4, Table S2). Specifically, after flowering, PN_cb_, PN_et_, and PN_cl_ increased significantly (Table S2). Moreover, there were strong positive correlations among PN_cb_, PN_et_, and PN_cl_ in photosynthetic components (all *p* < .01; Figure S5).

**FIGURE 4:**
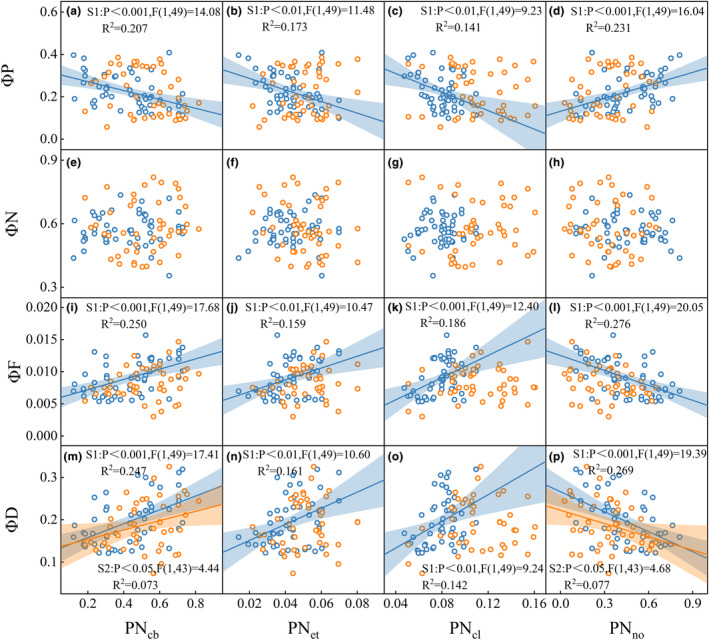
Relationship between non‐photochemical quenching (NPQ‐limited) energy partitioning: ΦP (photochemical yield) (a–d), ΦN (non‐photochemical quenching) (e–h), ΦF (fluorescence yield) (i–l), ΦD (nonradiative decay) (m–p) and leaf nitrogen allocation components: PN_cb_ (carboxylation system), PN_et_ (bioenergetic protein), PN_cl_ (light‐harvesting protein), PN_no_ (non‐photosynthetic). The colors of blue and orange correspond to vegetative growth and reproductive growth stages. Linear regression analysis was used to evaluate the relationship between variables, the shaded area represents the 95% confidence interval of the fitting line. The fitting line and 95% confidence interval are represented in the same color.

**FIGURE 5:**
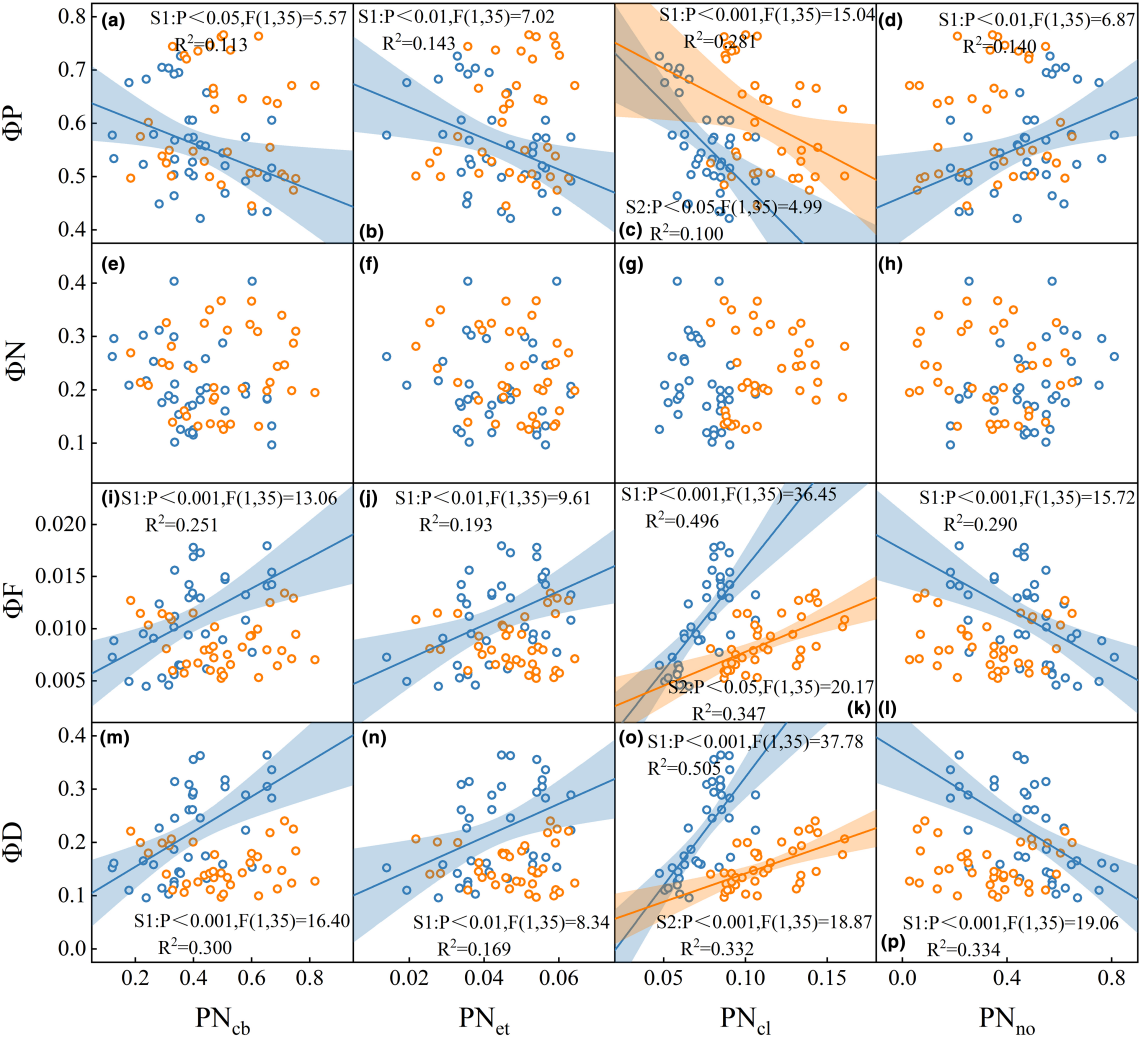
Relationship between photochemical quenching (PQ‐limited) energy partitioning: ΦP (photochemical yield) (a–d), ΦN (non‐photochemical quenching) (e–h), ΦF (fluorescence yield) (i–l), ΦD (nonradiative decay) (m–p) and leaf nitrogen allocation components: PN_cb_ (carboxylation system), PN_et_ (bioenergetic protein), PN_cl_ (light‐harvesting protein), PN_no_ (non‐photosynthetic). The colors of blue and orange correspond to vegetative growth and reproductive growth stages. Linear regression analysis was used to evaluate the relationship between variables, the shaded area represents the 95% confidence interval of the fitting line. The fitting line and 95% confidence interval are represented in the same color.

Leaf nitrogen allocation was found to have a significant impact on both photochemical yield (ΦP) and fluorescence yield (ΦF) (Figures 4 and 5). Specifically, the nitrogen investment proportion in photosynthetic components, including PN_cb_, PN_et_, and PN_cl_, exhibited a positive correlation with ΦF and a negative correlation with ΦP (Figures 4 and 5).

## 4 DISCUSSION

### 4.1 High leaf nitrogen investment in photosynthetic systems significantly increases fluorescence yield

Our research findings suggested that during vegetative growth stage of rice, increasing leaf nitrogen investment in photosynthetic system, specifically in the light‐harvesting protein (PN_cl_), carboxylation system (PN_cb_), and bioenergetic protein (PN_et_), lead to a significant enhancement in fluorescence yield (ΦF) (Figures 4 and 5). It can be attributed to the fact that nitrogen served as the primary raw material for the formation of photosynthetic components (Mu et al., 2016). Zhuang et al. (2021) found that the proportion of nitrogen allocated to light‐harvesting protein components mediated the relationship between *V*_cmax_ and chlorophyll content based on an observation along canopy profiles on a scaffold tower in a subtropical forest ecosystem of south China. Gao et al. (2018) reported that a decrease in nitrogen assimilation resulted in a reduction in Rubisco content, *V*_cmax_, and the NADPH and ATP demand generated by the Calvin cycle during the photo reaction stage. Han et al. (2022) established a predictive model for PSII SIF in relation to *V*_cmax_ and *J*_max_ by considering the balance between light and carbon reactions based on the 15 plant species measurements. They found that the product of PSII SIF and the fraction of open PSII reactions *q*_L_, which indicated the redox state of PSII, served as a robust and positive predictor for *V*_cmax_ and *J*_max_ (Han et al., 2022). Moreover, previous studies indicated that *V*_cmax_ have a strong correlation with C_ab_ (Croft et al., 2017; Houborg et al., 2013), resulting in a strong relationship with SIF (Yang et al., 2015). As a result, the variations in C_ab_ and N_mass_ contributed to a significant positive relationship between PN_cl_ and ΦF (Figures 3e and 4k). The investment proportion of leaf nitrogen in the light‐harvesting protein component (PN_cl_) played a crucial role in regulating energy partitioning, such as ΦP, ΦF and ΦD, during the vegetative growth stage (Figures 4 and 5). The initial step of photosynthesis involves the absorption of light by light‐harvesting complexes (Porcar‐Castell et al., 2014), with approximately 80% of nitrogen in C_3_ plant thylakoids being invested in light‐harvesting proteins in the form of chlorophyll (Amane et al., 2003; Mu et al., 2016). Furthermore, the chlorophyll content can be effectively predicted by satellite observations of SIF combined with the SCOPE model (Zhang et al., 2018; Zhang, Guanter, et al., 2014). In conclusion, our findings underscore the critical significance of leaf nitrogen allocation in understanding SIF dynamics.

Furthermore, the enhancement of nitrogen per leaf mass (N_mass_) was linked to higher photochemical yield (ΦP) along with a decrease in non‐photochemical quenching (ΦN) and fluorescence yield (ΦF) during the vegetative growth stages (Figure 3). Kumagai et al. (2009) observed that nitrogen deficiency caused the reductions in the photochemical yield (ΦP) and electron transport quantum yield of the rice flag leaf to varying extents. Similarly, decreased photochemical yield (ΦP) and reemitted excess energy through thermal dissipation or chlorophyll fluorescence were observed in C_4_ plant maize under low nitrogen with damage of PSII (Mu et al., 2017). However, Chen et al. (2003) reported that there was no significant difference in photochemical yield (ΦP) regardless of nitrogen treatment, which may be attributed to electron transfer chain saturation under high light intensity conditions (Zhang et al., 2018). Therefore, in addition to environmental changes, N_mass_ should also be taken into consideration when decoding PSII photochemical yield from SIF.

### 4.2 Growth variations of leaf energy partitioning and leaf nitrogen allocation

Interestingly, our results revealed significant variations in leaf energy partitioning across different growth stages (Tables 1 and 2). Specifically, ΦF was higher during the vegetative growth stage than that during the reproductive growth stage (Figure 1, Tables 1 and 2). After flowering, the growth variation in leaf energy partitioning can be attributed to the transfer of nutrients and the senescence of leaves. During the vegetative growth stage, nutrients and photosynthates primarily support leaf development, whereas during the reproductive growth stage, they are redirected towards the developing grains (Nuccio et al., 2015; Yu et al., 2015; Zhang et al., 2021). The onset of flowering is triggered when plants exhibit the peak nitrogen use efficiency (Guilbaud et al., 2015). During the reproductive growth stage, leaves undergo programmed senescence to allocate photosynthates and nutrients to reproductive tissues, as nitrogen use efficiency continues to decline (Tang et al., 2021; Yang et al., 1998). Leaf senescence at different levels resulted in the gradual emergence of nutritional stress (Abe et al., 2016; Anbari et al., 2015; Fan et al., 2019). Under nutritional stress, a decrease in ΦF accompanied by an increase in ΦN was observed in various crops, such as wheat, corn, bean, tomato, grape, and aloe (Bashir et al., 2021; Hazrati et al., 2016; Jahan et al., 2021; Luo et al., 2022; Sun et al., 2016; Sun, Gao, et al., 2018; Suzuki et al., 2021; Wang et al., 2018; Zhang et al., 2015). Similarly, increases in ΦN and decreases in ΦF have been observed in various crops under conditions of heat stress and chemical herbicide stress (Gautam et al., 2014; Li et al., 2017).

**TABLE 2:** Effects of different growth stages and nitrogen treatments on energy partitioning components.

		df	Residual df	ΦP		ΦN		ΦF		ΦD	
				*F*	*p*	*F*	*p*	*F*	*p*	*F*	*p*
ANOVA	Stage	1	174	0.677	.412	0.140	.709	10.305	**.002**	13.851	**.000**
	Nitrogen	2	174	0.079	.924	0.129	.879	0.278	.757	0.294	.745
	Nitrogen × Stage	2	174	0.091	.913	0.275	.760	0.261	.770	0.482	.619

*Note*: ΦD, nonradiative decay yield; ΦF, fluorescence yield; ΦN, non‐photochemical quenching yield; ΦP, photochemical yield. The results of significance tests (*p*‐values) for two‐way ANOVA, showing the effects of growth stages, nitrogen treatments, and their interactions, were presented in the table. Values were displayed in bold if they passed the significance test (*p* < .05). Degrees of freedom (df) and residual degrees of freedom (Residual df) were included alongside *p*‐values.

Our findings revealed that the average PN_cb_ of 0.49 throughout the whole growth cycle significantly exceeded the typical nitrogen allocation in C_3_ plant leaves, around 0.25. In contrast, PN_cl_ of 0.10 was notably lower than 0.18. Additionally, a relatively minor difference was observed in PN_et_, with values of 0.05 and 0.06, respectively (Mu & Chen, 2021). This is mainly because rice plants encounter excessive energy supply for substrate metabolism during field growth, such as high light and temperature conditions. In response, plants invest more in Rubisco, leading to significant increases in *V*_cmax_ and PN_cb_, but decreases in PN_cl_. However, *J*_max_ and PN_et_ remained unchanged (Katahata et al., 2007; Yin et al., 2019). Similarly, Yang et al. (2023) reported consistent patterns of *V*_cmax_, *J*_max_, and leaf nitrogen allocation in field observations of rice in subtropical regions. There were significant growth variations of leaf nitrogen allocation (Tables S3 and S4). Specifically, during the vegetative growth stage, the investment proportion of leaf nitrogen in photosynthetic system (PN_cb_, PN_et_, PN_cl_) was significantly lower compared to the reproductive growth stage. Conversely, the investment proportion of leaf nitrogen in non‐photosynthetic system exhibited the opposite trend. The observed growth variation in leaf nitrogen allocation could be attributed to a shift in investment strategy. During the vegetative growth stage, crops tended to optimize their energy utilization by increasing the investment proportion of non‐photosynthetic system to maximize leaf area index (LAI) and promote greater plant growth (Hirel et al., 2007; Pask et al., 2012). This strategy allows the crops to capture more light energy for enhanced photosynthetic efficiency and overall growth. In flowering time, the nitrogen absorption efficiency of leaves reaches its peak, crops canopy gradually closed and LAI reached a constant level, making the transition from crops own growth to reproduction of their offspring (Capelli et al., 2016; Hirel et al., 2007; Li et al., 2020; Pask et al., 2012; Spigler & Woodard, 2019; Züst et al., 2015). The nitrogen per leaf mass (N_mass_) and nitrogen absorption efficiency of leaves decrease continuously during the reproductive growth stage (Guilbaud et al., 2015). To maintain the continuous transport photosynthetic nitrogen and photosynthetic products to the grains (Mu & Chen, 2021; Pask et al., 2012), the investment proportion of nitrogen in the photosynthetic system increased (Tables S3 and S4). Nevertheless, it is inevitable that N_mass_ decreases after flowering and leaf senescence (Kamal et al., 2019; Pask et al., 2012; Xue et al., 2021).

### 4.3 Decoupling of photosystem fluorescence yield and leaf nitrogen allocation after flowering

Our findings revealed a decoupled relationship between photosystem fluorescence yield (ΦF) and leaf nitrogen allocation after flowering (Figures 4 and 5). First, the decoupling was attributed to the senescence of crops after flowering. Herbs transfer nutrients into seeds and fruits by aging and sacrificing leaves from the bottom to the top of the crown (Yang et al., 1998). During the leaf senescence process, leaf cells have undergone great changes in cell metabolism, structure, and gene expression, especially in chloroplast degradation (Pottier et al., 2014; Xue et al., 2021). Second, the decoupling was attributed to nitrogen stress of crops after flowering (Mu & Chen, 2021). When the peak of plant nitrogen absorption efficiency triggered flowering, the plant appeared to have different degrees of nitrogen deficiency (Guilbaud et al., 2015; Pask et al., 2012). Furthermore, our findings revealed an inconsistent relationship between ΦF and ΦP before and after flowering (Figure 2). Li et al. (2020) reported significant differences in chlorophyll fluorescence (SIF) and photosynthetic capacity (*V*_cmax_) before and after flowering. Additionally, Yang et al. (2018) observed that canopy ΦF were high before flowering, and remained low in mature leaves. These findings highlight the crucial role of flowering in regulating seasonal dynamics of photosystem fluorescence yield and leaf nitrogen allocation.

## 5 CONCLUSION

We quantified the relationship between energy partitioning and nitrogen allocation in leaves throughout the growing season in rice in the subtropical region of China. Fluorescence yield (ΦF) during the vegetative growth stage has a strong ability to track the investment proportion of leaf nitrogen in photosynthetic system, such as light‐harvesting protein, bioenergetic protein, and carboxylation system. There were significant differences in energy partitioning and nitrogen allocation in the photosynthetic system before and after flowering. Our findings highlight the crucial role of phenological factors in exploring seasonal photosynthetic dynamics and carbon fixation of ecosystems.

## AUTHOR CONTRIBUTIONS

**Duwei Zhong:** Conceptualization (equal); data curation (equal); formal analysis (equal); investigation (equal); methodology (equal); project administration (equal); software (equal); supervision (equal); validation (equal); visualization (equal); writing – original draft (equal); writing – review and editing (equal). **Yonggang Chi:** Conceptualization (equal); data curation (equal); formal analysis (equal); funding acquisition (equal); investigation (equal); methodology (equal); project administration (equal); resources (equal); software (equal); supervision (equal); validation (equal); visualization (equal); writing – original draft (equal); writing – review and editing (equal). **Jianxi Ding:** Conceptualization (equal); data curation (equal). **Ning Zhao:** Conceptualization (equal); data curation (equal). **Linhui Zeng:** Data curation (equal). **Pai Liu:** Data curation (equal); investigation (equal). **Zhi Huang:** Conceptualization (equal); data curation (equal); formal analysis (equal); investigation (equal); methodology (equal); resources (equal); supervision (equal). **Lei Zhou:** Conceptualization (equal); data curation (equal); formal analysis (equal); funding acquisition (equal); investigation (equal); methodology (equal); project administration (equal); resources (equal); software (equal); supervision (equal); validation (equal); visualization (equal); writing – original draft (equal); writing – review and editing (equal).

## CONFLICT OF INTEREST STATEMENT

The authors declare that they have no known competing financial interests or personal relationships that could have appeared to influence the work reported in this paper.

## Supporting information


Appendix S1

Appendix S2

## Data Availability

The data associated with this analysis are stored on the Dryad Digital Repository: https://doi.org/10.5061/dryad.3xsj3txmz. The following link initiates downloading only data files when the manuscript is in peer review and the dataset has not been published: https://datadryad.org/stash/share/clSW0qWcIMH2i7X30iIP_xUzAMN‐vFbZvlMe4Q9Y8‐w.
