# Supplementary material for: Decoupling of nitrogen allocation and energy partitioning in rice after flowering
Source: Ecol Evol. 2024 Apr 15;14(4):e11297. doi: 10.1002/ece3.11297 (PMC11017445; doi:10.1002/ece3.11297)
Supplement: Supplementary file 1 — Appendix S1 [file ECE3-14-e11297-s002.docx]

**Appendix**

**Note S1.** **Calculation of Photosynthetic Active Radiation Absorbed by Leaves**

The proportion of absorbed photosynthetic radiation (f_PAR_) was calculated using the f_PAR_-NDVI linear model as follows: for farmland, NDVI_max_=0.926, NDVI_min_=0.13, f_PARmax_=0.95, f_PARmin_=0.001.

$$\begin{aligned} NDVI=\frac{NIR-RED}{NIR+RED} \end{aligned}(S1)$$

$$\begin{aligned} f_{PAR}=\frac{\left( \mathrm{NDVI}-\mathrm{NDVI}_{min} \right)\left( f_{PARmax}-f_{PARmin} \right)}{\mathrm{NDVI}_{max}-\mathrm{NDVI}_{min}}-f_{PARmin} \end{aligned}(S2)$$

$$\begin{aligned} \mathrm{APAR}_{leaf}=\mathrm{PAR}_{leaf}\times f_{PAR} \end{aligned}(S3)$$

**Note S2.** **Calculation of nitrogen investment in photosynthesis**

The investment of leaf nitrogen in photosynthesis was divided into three components, including carboxylation system (N_cb_, a protein used for carboxylation reactions in the Calvin cycle); bioenergetic protein (N_et_ refers to proteins used for electron transport) and light-harvesting protein component (N_cl_ refers to light harvesting proteins used in photosystem and other light-harvesting pigment protein complexes).

$$\begin{aligned} N_{cb}=\frac{V_{cmax25}}{6.25\times V_{cr}} \end{aligned}(S4)$$

$$\begin{aligned} N_{et}=\frac{J_{max25}}{8.06\times J_{mc}} \end{aligned}(S5)$$

$$\begin{aligned} N_{cl}=\frac{C_{ab}}{C_{B}} \end{aligned}(S6)$$

$$\begin{aligned} V_{cmax25}\left( J_{max25} \right)=\frac{\left( 1+e^{\left( \frac{\Delta ST_{0}-{\Delta H}_{d}}{RT_{0}} \right)} \right)e^{\frac{{\Delta H}_{0}}{RT_{0}}\left( 1-\frac{T_{0}}{T} \right)}}{1+e^{\frac{\Delta ST-{\Delta H}_{d}}{RT}}} \end{aligned}(S7)$$

The value of 6.25 is the conversion coefficient of rubisco enzyme to nitrogen and 8.06 is the number of cytochrome per gram of nitrogen in the bioenergy conversion carrier. V_cr_ represents the CO_2_ carboxylation activity of the unit rubisco enzyme with a value of 20.78 μmol CO_2_ (g rubisco) ^−1^ s^−1^ at 25 ℃; J_mc_ represents the number of electrons transmitted per second by cytochrome with a value of 155.65 μmol electrons (μmol Cyt f) ^−1^ s^−1^ at 25 ℃. C_ab_ is the chlorophyll content (mmol g^−1^); C_b_ is the content of the chlorophyll-protein complex with a value of 2.15 mmol g^−1^. R is the gas constant with a value of 8.314 J mol^-1^ K^-1^ ([Bernacchi et al., 2004](#_ENREF_11)). T represents leaf temperature in Kelvin (K), and T_0_ is the reference temperature with a value of 298.15 K. ΔH_a_, ΔH_d_, ΔS, activation energy, deactivation energy, entropy, and proportionality constant have the following values when calculating V_cmax25_: 74000 J mol^-1^, 203000 J mol^-1^, 645 J K^-1^ mol^-1^, and 32.9, respectively ([Niinemets and Tenhunen, 1997](#_ENREF_53)). Conversely, when calculating J_max25_, their values are 24100 J mol^-1^, 564150 J mol^-1^, 1810 J K^-1^ mol^-1^, and 14.77 ([Kattge et al., 2010](#_ENREF_36); [Medlyn et al., 2010](#_ENREF_45)).

**References**

1. Bernacchi, C. J., Singsaas, E. L., Pimentel, C., Portis, A. R., Jr., & Long, S. P. (2004). Improved temperature response functions for models of rubisco‐limited photosynthesis. Plant, Cell & Environment, 24(2), 253–259, https://doi.org/10.1111/j.1365-3040.2001.00668.x.
2. Niinemets, Ü., & Tenhunen, J. D. (1997). A model separating leaf structural and physiological effects on carbon gain along light gradients for the shade‐tolerant species Acer saccharum. Plant, Cell and Environment, 20(7), 845–866, https://doi.org/10.1046/j.1365-3040.1997.d01-133.x.
3. Kattge, J., & Knorr, W. J. P. C. (2010). Temperature acclimation in a biochemical model of photosynthesis: A reanalysis of data from 36 species. Plant, Cell & Environment, 30(9), 1176–1190, https://doi.org/10.1111/j.1365-3040.2007.01690.x.
4. Medlyn, B. E., Dreyer, E., Ellsworth, D., Forstreuter, M., Harley, P. C., Kirschbaum, M. U. F., le Roux, X., Montpied, P., Strassemeyer, J., Walcroft, A., Wang, K., & Loustau, D. (2010). Temperature response of parameters of a biochemically based model of photosynthesis. II. A review of experimental data. Plant, Cell & Environment, 25(9), 1167–1179, https://doi.org/10.1046/j.1365-3040.2002.00891.x.
