# Supplementary material for: Decoupling of nitrogen allocation and energy partitioning in rice after flowering
Source: Ecol Evol. 2024 Apr 15;14(4):e11297. doi: 10.1002/ece3.11297 (PMC11017445; doi:10.1002/ece3.11297)
Supplement: Supplementary file 2 — Appendix S2 [file ECE3-14-e11297-s001.docx]

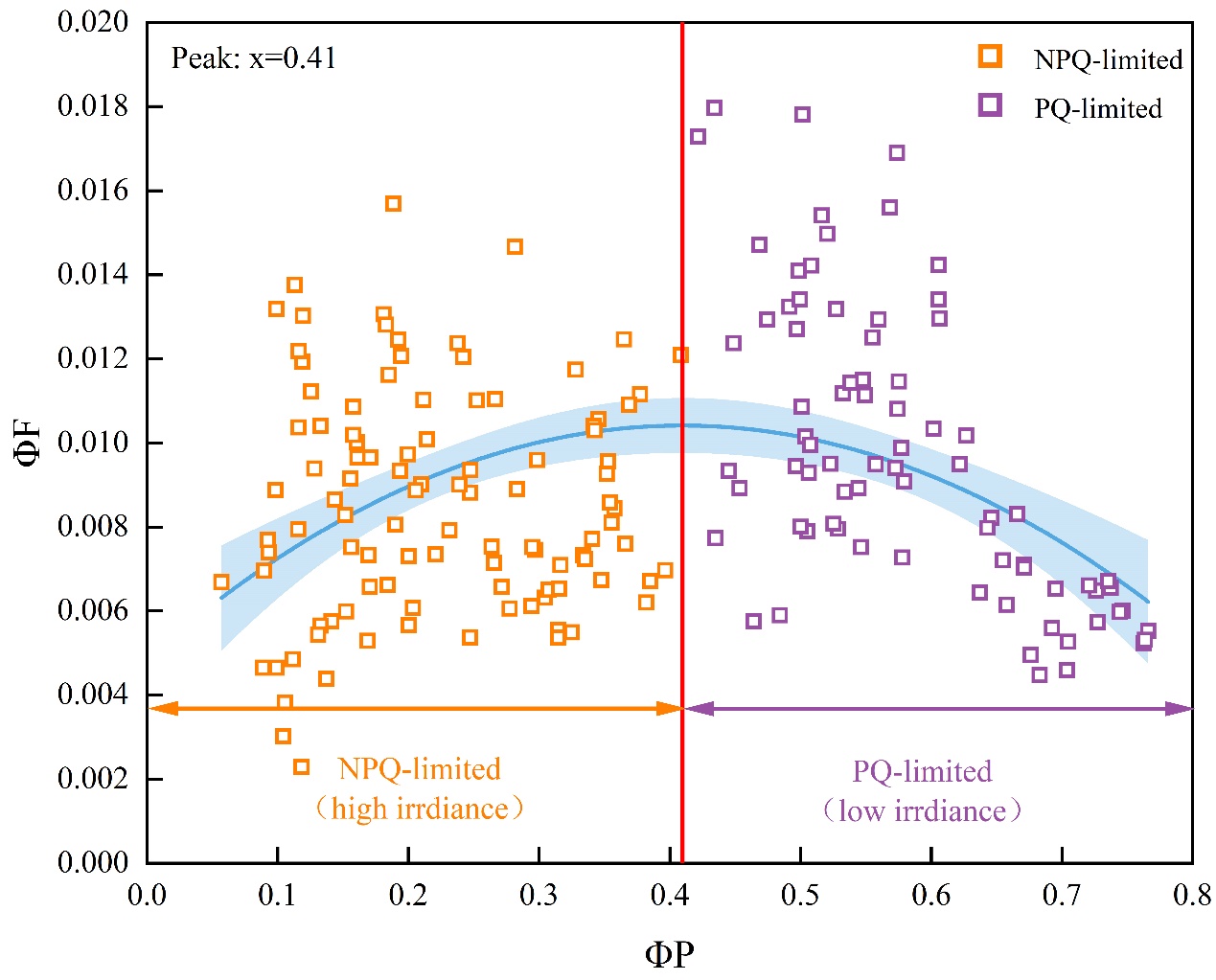


**Figure S1** Relationship between ΦP (photochemical yield) and ΦF (fluorescence yield). Using second-order polynomial fitting to evaluate the relationship between ΦP and ΦF, the shaded area is the 95% confidence interval of second-order polynomial fitting. Breakpoints (dashed lines) were identified as the value of ΦP at which the first derivative (i.e., slope) equaled zero and the slope switched from positive to negative. The left side of the breakpoint indicates that ΦF is limited by non-photochemical quenching (NPQ-limited) (orange), while the right side indicates that ΦF is limited by photochemical quenching (PQ-limited) (purple).


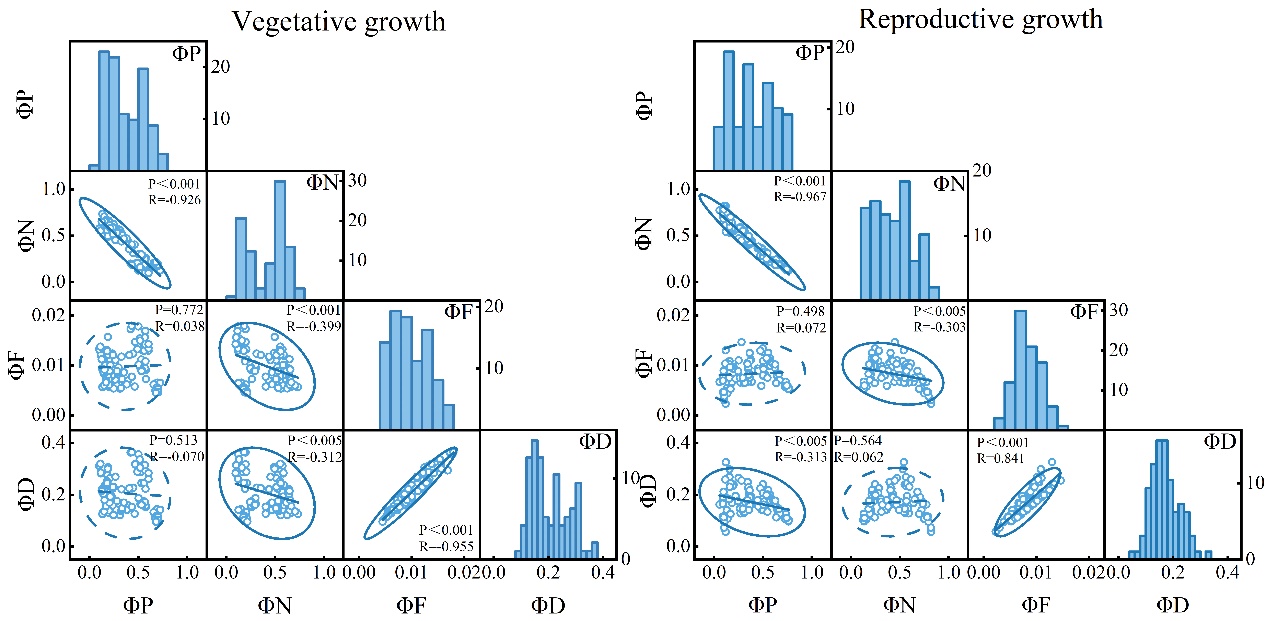


**Figure S2** ΦP: photochemical yield, ΦN: non-photochemical quenching yield, ΦF: fluorescence yield, ΦD: nonradiative decay yield. Correlation scatter matrix diagram of energy partitioning at different growth stages. Below the diagonal is the data scatter, on the left is the scatter scale, the ellipse is a 95% confidence ellipse. Diagonal represents the frequency distribution histogram of the data, with the left and right graphs displaying the frequency distribution of energy partitioning for the first five and last five measurements, respectively. The right-side scale represents the frequency scale of the histogram. P is the significance level (p<0.05), and R is the correlation coefficient.


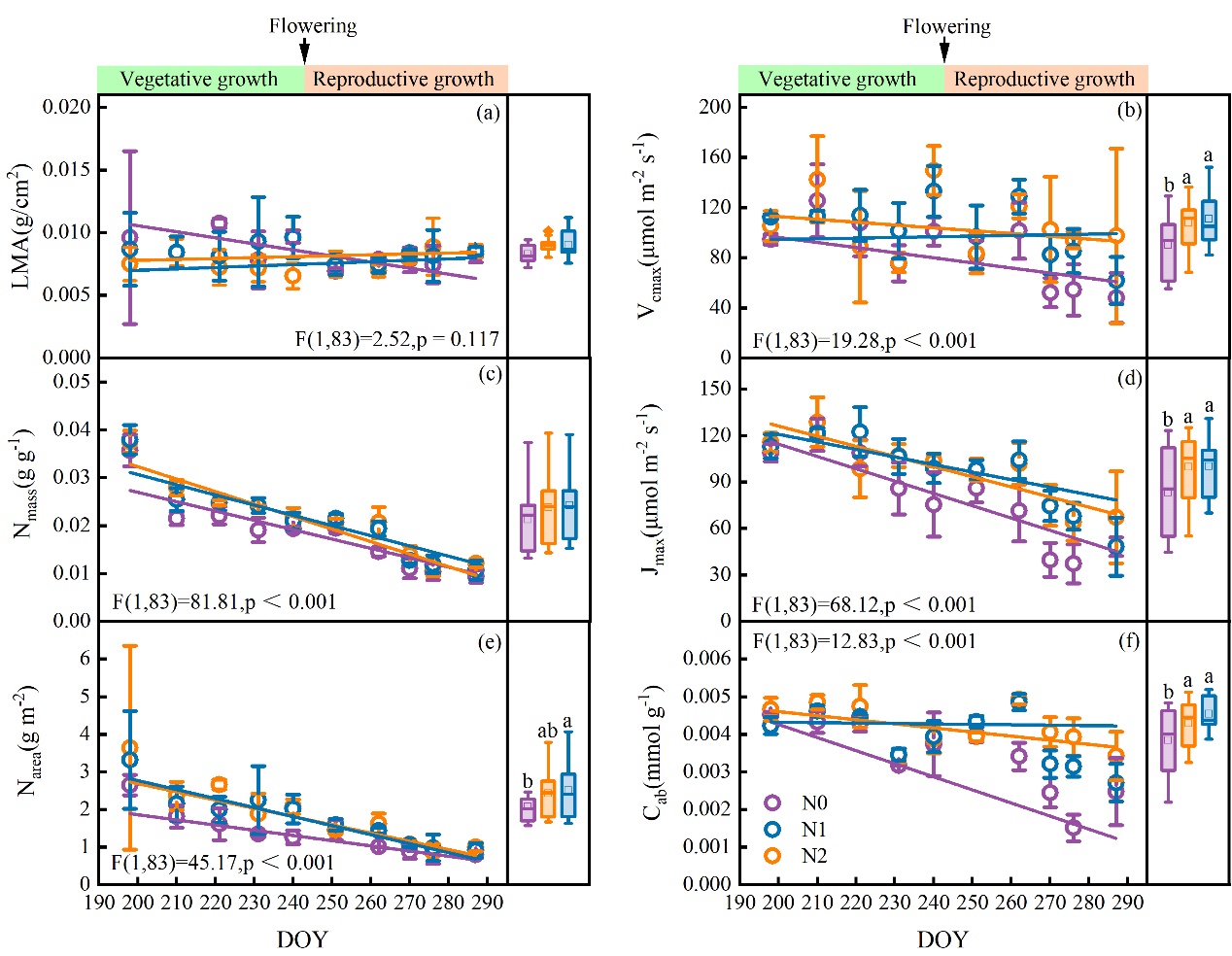


**Figure S3** The growth variation of leaf mass per area (LMA) (a), maximum carboxylation rate (V_cmax_) (b), nitrogen per leaf mass (N_mass_) (c), maximum photoelectron transfer rate (J_max_) (d), nitrogen content based on leaf area (N_area_) (e), and leaf chlorophyll content (C_ab_) (f) under nitrogen treatments. The point and error bar line reflect the average value (± SE) of the three duplicates, and the straight line is a trend line fitted linearly. The P-value represents the significance test result of the one-way ANOVA of variance for growth stages. Purple, blue and orange correspond to Low-N (N0), Middle-N (N1) and High-N (N2) treatment groups, respectively. The box graph on the right shows the overall distribution of data in different processing groups, with the letters "a" and "b" indicated above, indicating significant differences among different nitrogen treatments based on the one-way ANOVA of variance. Different lowercase letters indicate significant difference between different nitrogen treatments.


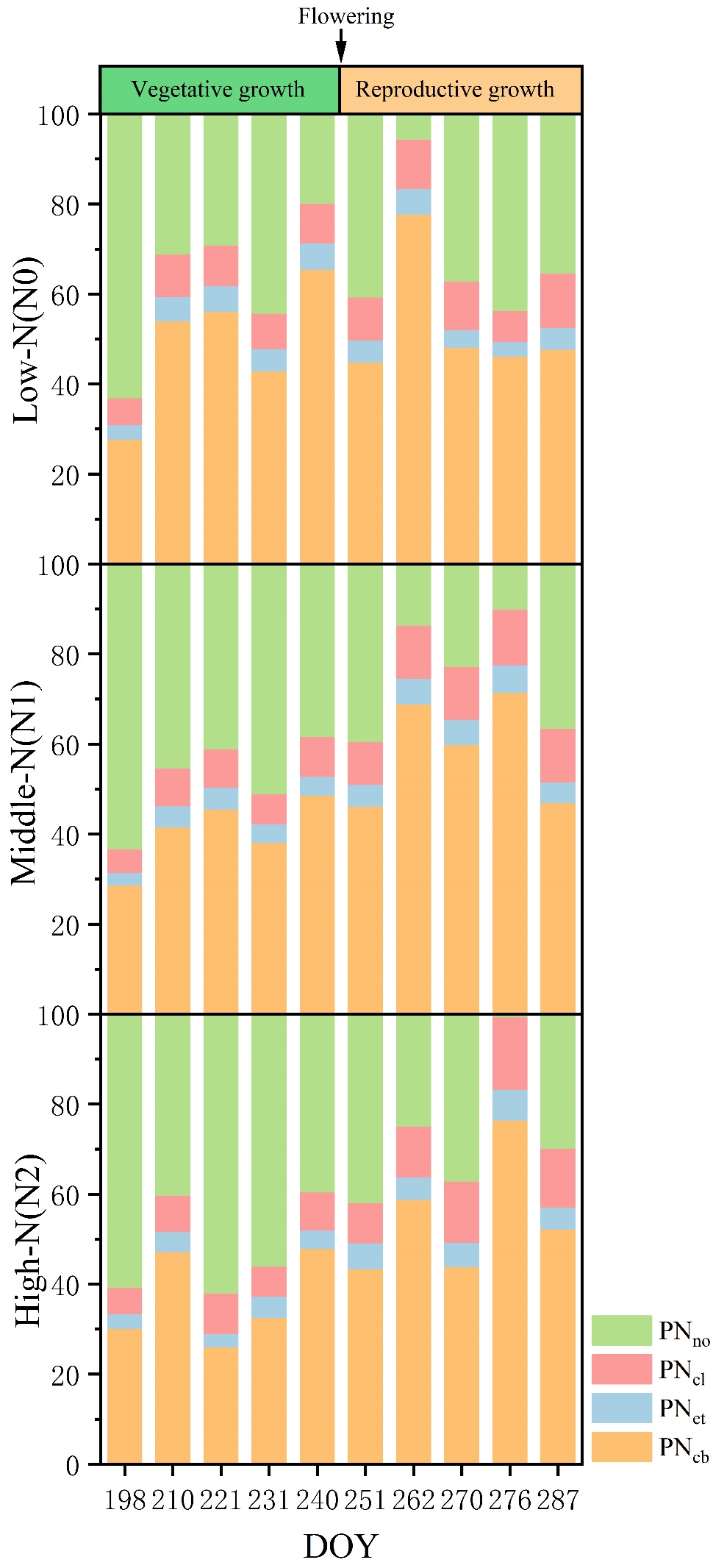


**Figure S4** Seasonal variations in leaf nitrogen allocation percentage (PN) for the different pathways: carboxylation system (PN_cb_, orange), bioenergetic protein (PN_et_, sky blue), light-harvesting protein components (PN_cl_, magenta), and non-photosynthetic components (PN_no_, light green).


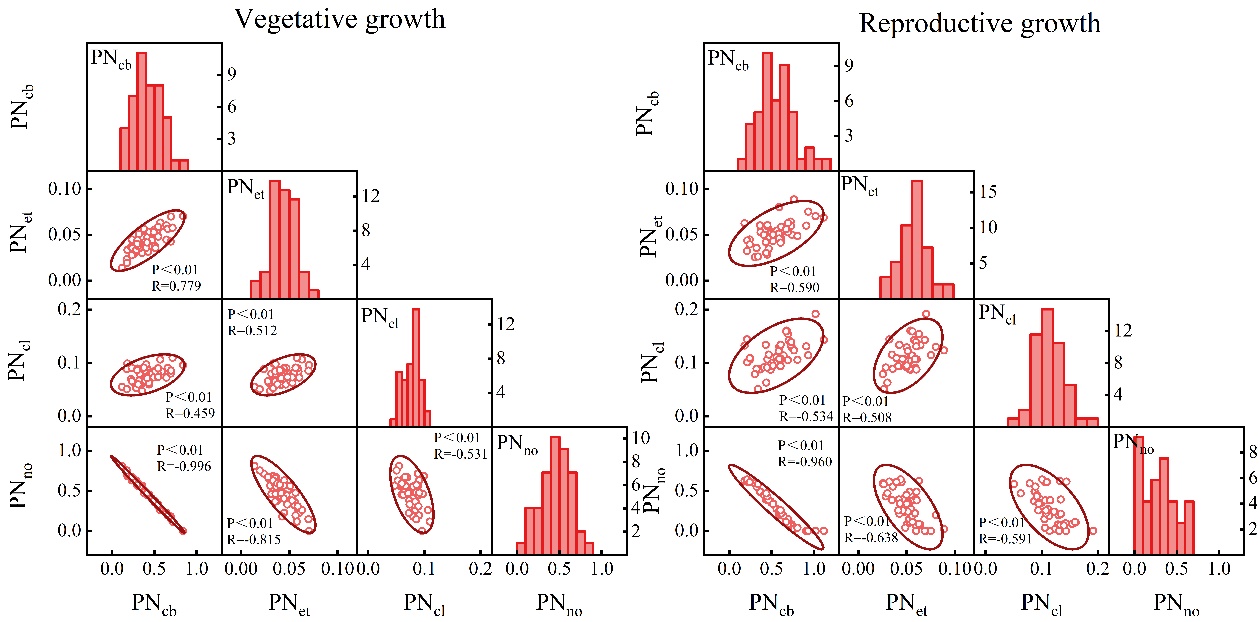


**Figure S5** PN_cb_: the proportion of leaf nitrogen allocated to carboxylation system component, PN_et_: the proportion of leaf nitrogen allocated to bioenergetic protein component, PN_cl_: the proportion of leaf nitrogen allocated to light-harvesting protein component, PN_no_: the proportion of leaf nitrogen allocated to non-photosynthetic component. Scatter matrix of nitrogen allocation at different growth stages. Below the diagonal is the data scatter, on the left is the scatter scale, the ellipse is a 95% confidence ellipse. Diagonal represents the frequency distribution histogram of the data, with the left and right graphs displaying the frequency distribution of nitrogen allocation for the first five and last five measurements, respectively. The right-side scale represents the frequency scale of the histogram. P is the significance level (p<0.05), and R is the correlation coefficient.


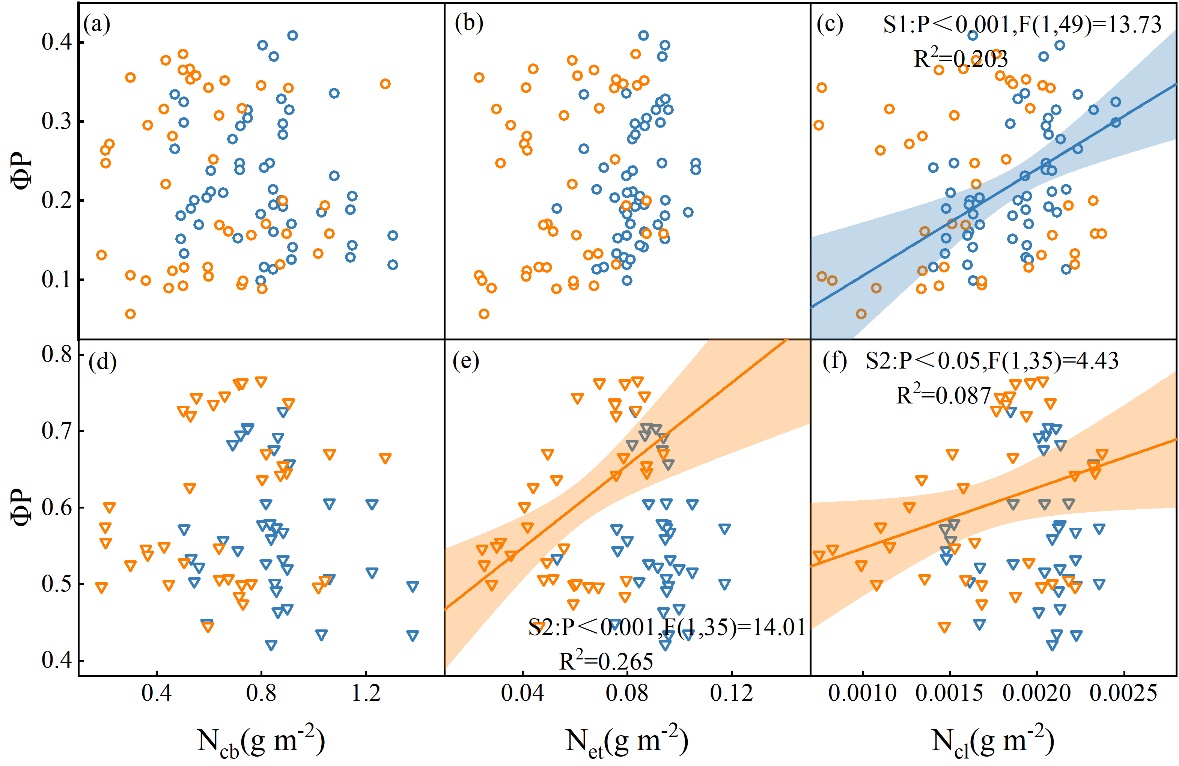


**Figure S6** Relationship between ΦP (photochemical yield) and the investment of leaf nitrogen in photosynthetic components: N_cb_ (carboxylation system), N_et_ (bioenergetic protein), N_cl_ (light-harvesting protein). The colors of blue and orange correspond to vegetative growth and reproductive growth stages. Hollow circles and hollow triangles represent non-photochemical quenching (NPQ-limited) (a, b, c) and photochemical quenching (PQ-limited) (d, e, f), respectively. Linear regression analysis was used to evaluate the relationship between variables, the shaded area represents the 95% confidence interval of the fitting line. The fitting line and 95% confidence interval are represented in the same color.

**Table S1** Means and standard errors of physiological parameters under different growth stages and nitrogen treatments.

| Stage | Nitrogen | LMA | N_mass_ | N_area_ | V_cmax_ | J_max_ | C_ab_ |
| --- | --- | --- | --- | --- | --- | --- | --- |
| S1 | N0 | 0.007 (b) | 0.024 (a) | 1.74 (b) | 100.83 (a) | 99.86 (b) | 0.004 (a) |
|  | N1 | 0.009 (ab) | 0.027 (a) | 2.35 (ab) | 114.76 (a) | 112.42 (a) | 0.004 (a) |
|  | N2 | 0.009 (a) | 0.027 (a) | 2.53 (a) | 112.42 (a) | 110.76(ab) | 0.004 (a) |
| S2 | N0 | 0.008 (a) | 0.013 (a) | 1.02 (a) | 67.62 (b) | 56.50 (b) | 0.003 (b) |
|  | N1 | 0.008 (a) | 0.015 (a) | 1.20(a) | 91.02 (a) | 78.58 (a) | 0.004 (a) |
|  | N2 | 0.008 (a) | 0.016 (a) | 1.20 (a) | 99.69 (a) | 81.20 (a) | 0.004 (a) |

S1: vegetative growth stage, S2: reproductive growth stage, LMA: leaf mass per area, N_mass_: nitrogen per leaf mass, N_area_: nitrogen content based on leaf area, V_cmax_: maximum carboxylation rate, J_max_: maximum photoelectron transfer rate, C_ab_: leaf chlorophyll content. Values are means (n = 15). In the same column within a specific growth stage, different lowercase letters indicate significant differences between nitrogen treatments as determined by ANOVA (p<0.05).

**Table S2** Effects of different growth stages and nitrogen treatments on physiological parameters.

|  |  |  |  | LMA | | N_mass_ | | N_area_ | | V_cmax_ | | J_max_ | | C_ab_ | |
| --- | --- | --- | --- | --- | --- | --- | --- | --- | --- | --- | --- | --- | --- | --- | --- |
|  |  | DF | Residual DF | F | P | F | P | F | P | F | P | F | P | F | P |
| ANOVA | Stage | 1 | 81 | 2.560 | 0.122 | 85.859 | **0.000** | 48.209 | **0.000** | 20.139 | **0.000** | 73.760 | **0.000** | 15.209 | **0.000** |
|  | Nitrogen | 2 | 81 | 1.629 | 0.217 | 3.025 | 0.059 | 3.750 | **0.032** | 2.807 | 0.070 | 4.396 | **0.015** | 8.589 | **0.000** |

LMA: leaf mass per area, N_mass_: nitrogen per leaf mass, N_area_: nitrogen content based on leaf area, V_cmax_: maximum carboxylation rate, J_max_: maximum photoelectron transfer rate, C_ab_: leaf chlorophyll content. The results of significance tests (p-values) for two-way ANOVA, showing the effects of growth stages and nitrogen treatments were presented in the table. Values were displayed in bold if they passed the significance test (p < 0.05). Degrees of freedom (DF) and residual degrees of freedom (Residual DF) were included alongside p-values.

**Table S3** Means and standard errors of nitrogen allocation components under different growth stages and nitrogen treatments.

| Stage | Nitrogen | PN_cb_ | PN_et_ | PN_cl_ | PN_no_ |
| --- | --- | --- | --- | --- | --- |
| S1 | N0 | 0.49±0.18(a) | 0.05±0.00(a) | 0.08±0.02(a) | 0.37±0.21(b) |
|  | N1 | 0.41±0.14(ab) | 0.04±0.01 (b) | 0.08±0.02(a) | 0.48±0.16(ab) |
|  | N2 | 0.37±0.17(b) | 0.04±0.01(b) | 0.08±0.01(a) | 0.52±0.18(a) |
| S2 | N0 | 0.53±0.20(a) | 0.05±0.02 (b) | 0.10±0.03(b) | 0.33±0.23(a) |
|  | N1 | 0.59±0.18(a) | 0.05±0.013(ab) | 0.11±0.02(ab) | 0.25±0.19(a) |
|  | N2 | 0.55±0.27 (a) | 0.06±0.01 (a) | 0.13±0.03(a) | 0.27±0.30(a) |

S1: vegetative growth stage, S2: reproductive growth stage, PN_cb_: the proportion of leaf nitrogen allocated to carboxylation system component, PN_et_: the proportion of leaf nitrogen allocated to bioenergetic protein component, PN_cl_: the proportion of leaf nitrogen allocated to light-harvesting protein component, PN_no_: the proportion of leaf nitrogen allocated to non-photosynthetic component. Values are means ± SE (n = 15). In the same column within a specific growth stage, different lowercase letters indicate significant differences between nitrogen treatments as determined by ANOVA (p<0.05).

**Table S4** Effects of different growth stages and nitrogen treatments on nitrogen allocation components.

|  |  |  |  | PN_cb_ | | PN_et_ | | PN_cl_ | | PN_no_ | |
| --- | --- | --- | --- | --- | --- | --- | --- | --- | --- | --- | --- |
|  |  | DF | Residual DF | F | P | F | P | F | P | F | P |
| ANOVA | Stage | 1 | 79 | 7.341 | **0.008** | 5.486 | **0.022** | 62.081 | **0.000** | 12.441 | **0.001** |
|  | Nitrogen | 2 | 79 | 1.010 | 0.369 | 0.454 | 0.637 | 1.175 | 0.314 | 0.701 | 0.499 |
|  | Nitrogen × Stage | 2 | 79 | 0.232 | 0.794 | 2.595 | 0.081 | 3.727 | **0.028** | 0.551 | 0.578 |

PN_cb_: the proportion of leaf nitrogen allocated to carboxylation system component, PN_et_: the proportion of leaf nitrogen allocated to bioenergetic protein component, PN_cl_: the proportion of leaf nitrogen allocated to light-harvesting protein component, PN_no_: the proportion of leaf nitrogen allocated to non-photosynthetic component. The results of significance tests (p-values) for two-way ANOVA, showing the effects of growth stages, nitrogen treatments, and their interactions, were presented in the table. Values were displayed in bold if they passed the significance test (p < 0.05). Degrees of freedom (DF) and residual degrees of freedom (Residual DF) were included alongside p-values.
